# Going green for perioperative hemodynamic monitoring: a golden opportunity for middle-income countries

**DOI:** 10.62675/2965-2774.20250379

**Published:** 2025-05-13

**Authors:** Frederic Michard, Mario Diego Teles Correia, Flavio Eduardo Nacul, Vinícius Caldeira Quintão

**Affiliations:** 1 MiCo Vallamand Switzerland MiCo - Vallamand, Switzerland.; 2 Intensive Care Unit Real Hospital Português Recife PE Brazil Intensive Care Unit, Real Hospital Português - Recife (PE), Brazil.; 3 Universidade Federal do Rio de Janeiro Rio de Janeiro RJ Brazil Universidade Federal do Rio de Janeiro - Rio de Janeiro (RJ), Brazil.; 4 Universidade de São Paulo Hospital das Clínicas Faculdade de Medicina São Paulo SP Brazil Hospital das Clínicas, Faculdade de Medicina, Universidade de São Paulo - São Paulo (SP), Brazil.

## INTRODUCTION

The question of environmentally sustainable perioperative medicine represents a new challenge in an era of cost constraints and climate crisis.^(1,2)^ If global healthcare were a country, it would be part of the top 10 largest carbon emitters on the planet.^(2)^ The European Society of Anesthesiology and Intensive Care (ESAIC), the World Federation of Societies of Anesthesiologists (WFSA), and the European Society of Intensive Care Medicine (ESICM) recently called for action to promote and favor green solutions in anesthesia and critical care.^(3-5)^

## THE PLASTIC WASTE BURDEN

The carbon footprint of operating rooms and intensive care units is dominated by the energy used for heating, ventilation, and air conditioning.^(6)^ Nevertheless, the amount of waste produced daily is highly significant and dominated by plastics.^(2,7)^ Producing 1kg of plastic generates approximately 3kg of carbon dioxide.^(8)^ This estimate considers the entire life cycle of plastic, from raw materials extraction to waste disposal. In addition, micro- and nano-plastics are now everywhere in our environment. Humans breathe, drink, and eat plastic; as a result, many of our organs contain plastic.^(9)^ Consequences on human health remain largely unknown but are unlikely favorable. For example, in patients undergoing carotid surgery, a recent study found that the presence of micro-plastics and nano-plastics in atheroma was not only standard (60% of the cases) but also associated with a significant increase in cardiovascular adverse events.^(10)^

## LITTLE BROOKS MAKE GREAT RIVERS

Multiple solutions have been proposed to decrease the carbon footprint of anesthesia and critical care. For example, reusable laryngoscope blades and pulse oximetry sensors exemplify circular economy principles, contrasting with the culture of single-use products. It has also been proposed to reduce the use of volatile anesthetics, which are potent greenhouse gases.^(7)^ Indeed, if the global warming potential of carbon dioxide is 1, then it is 144 for sevoflurane, 539 for isoflurane, and 2,540 for desflurane. Thus, many hospitals have banned desflurane from operating rooms. A single green initiative may have a limited impact on hospital's carbon footprint; however, the cumulative effect of multiple marginal gains can lead to significant improvements.

Regarding hemodynamic monitoring, when pulmonary artery pressure monitoring or extravascular lung water measurements are deemed useful (e.g., in patients with severe pulmonary hypertension and pulmonary edema, respectively), pulmonary and transpulmonary thermodilution techniques may still be justified. However, several alternative methods exist to monitor cardiac output. A recent survey^(11)^ showed that 92% of European anesthesiologists use uncalibrated pulse contour or pulse wave analysis (PWA) techniques to monitor cardiac output during the perioperative period, and meta-analyses suggest they help guide fluid management and decrease postoperative morbidity.^(12)^

**Figure 1:**
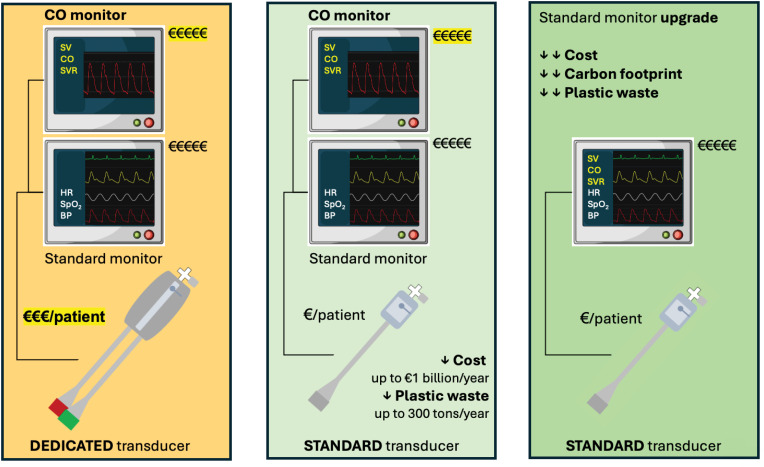
Improving sustainability and affordability of pulse wave analysis techniques for cardiac output monitoring.

There are two categories of uncalibrated PWA techniques: some require a dedicated pressure transducer (e.g., FloTrac or Acumen IQ from Becton Dickinson, USA), whereas others work with any standard pressure transducer.^(13)^ Dedicated pressure transducers are bigger, include two cables, and contain more plastic than standard pressure transducers (Figure 1). In addition, they are typically packaged in rigid plastic blisters, whereas standard transducers usually come in soft and light plastic and paper bags. For these reasons, PWA techniques working with standard transducers are now known as green cardiac output monitoring techniques.^(13,14)^ In a nationwide French study,^(14)^ the use of green PWA techniques for cardiac output monitoring during high-risk surgery was estimated to reduce plastic waste by 25 tons per year and carbon dioxide emissions between 65 and 83 tons per year. These are underestimations of the environmental advantages of green PWA techniques because the packaging was not considered in the carbon footprint evaluation. A more recent estimation,^(15)^ extended to all European countries and considering the packaging, suggested that green PWA techniques may decrease plastic waste and carbon dioxide emissions by 300 and 1,000 tons per year, respectively.

Left, dedicated cardiac output monitor + dedicated pressure transducer; middle, dedicated cardiac output monitor + standard pressure transducer; right, standard monitor integrating a pulse wave analysis algorithm + standard pressure transducer.

SV - stroke volume; CO - cardiac output; SVR - systemic vascular resistance; HR - heart rate; SpO^2^ - oxygen saturation; BP - blood pressure.

## REDUCING PLASTIC WASTE, BUT AT WHAT COST?

Before adopting green PWA techniques for perioperative cardiac output monitoring, two factors must be considered: first, that they are as reliable as other PWA techniques, and second, that they are not more expensive.

Several head-to-head comparison studies suggested that green PWA techniques have higher accuracy, precision, and concordance rates (quantifying the ability to track changes in cardiac output). For instance, Romagnoli et al.^(16)^ reported a two-to-three times lower percentage error with the MostCareUp (Vygon, France) than with the FloTrac system. Hadian et al.^(17)^ reported a bias and limits of agreement that were two times lower and narrower, respectively, with the LiDCOrapid (Masimo, USA) than with the FloTrac system. Recently, Mukkamala et al.^(18)^ reported a much lower bias (0.20 *versus* 0.94L/minute) with the Argos (Retia, USA) than with the FloTrac system. Of note, the MostCareUp, the LiDCOrapid, and the Argos systems are all green PWA techniques working with standard pressure transducers. In other words, according to data published so far, adopting green PWA techniques does not imply any sacrifice in terms of accuracy and precision. However, more studies comparing green and non-green PWA techniques would be helpful to confirm this is the case in most clinical situations.

A recent survey conducted in high-income countries showed that most anesthesiologists want to monitor cardiac output in high-risk surgical patients. However, most do not because "the equipment is unavailable and too expensive".^(11)^ Therefore, the cost of hemodynamic monitoring systems is a significant limitation to their clinical adoption. Interestingly, green PWA techniques also have the advantage of being much less expensive than other PWA techniques. In the aforementioned nationwide French comparison study,^(14)^ the use of green PWA techniques resulted in annual savings of €67 million, an amount equivalent to the annual salaries of over 2,000 French nurses or the purchase of more than 10,000 pocket ultrasound devices. In the European evaluation,^(15)^ adopting green PWA techniques was associated with annual savings ranging from €300 million to €1.2 billion. This broad range is attributed to the significant variability in transducer costs from one country to another. Therefore, a similar cost evaluation would benefit middle-income countries, including Brazil, where the cost of imported disposable products may sometimes be paradoxically higher than in high-income European countries. This analysis would allow for more precise quantification of the economic benefits of the transition to green PWA techniques for perioperative cardiac output monitoring.

Finally, the next desirable move from the medical device industry is integrating PWA algorithms into multi-variable regular bedside monitors, obviating the need for specific cardiac output monitors. Indeed, as illustrated in figure 1, such innovation would further reduce the cost and carbon footprint of cardiac output monitoring.

## CONCLUSION

Anesthesiologists favor uncalibrated pulse wave analysis techniques for continuous cardiac output monitoring in high-risk surgical patients. Several studies suggest that green pulse wave analysis techniques are as reliable as those requiring dedicated pressure transducers. They can minimize plastic waste and related carbon dioxide emissions and significantly reduce hospital costs. Such an evolution promises to improve the access to cardiac output monitoring, particularly in resource-limited settings.
